# Authenticated Public Key Elliptic Curve Based on Deep Convolutional Neural Network for Cybersecurity Image Encryption Application

**DOI:** 10.3390/s23146589

**Published:** 2023-07-21

**Authors:** Esam A. A. Hagras, Saad Aldosary, Haitham Khaled, Tarek M. Hassan

**Affiliations:** 1 Faculty of Engineering, Delta University for Science and Technology, Gamasa 35712, Egypt; esam.hagras@deltauniv.edu.eg; 2 Department of Computer Science, Community College, King Saud University, Riyadh 11437, Saudi Arabia; saldosary@ksu.edu.sa; 3 Department of Electronics and Communications, School of Engineering, Edith Cowan University, Perth, WA 6027, Australia; h.khaled@ecu.edu.au

**Keywords:** deep convolutional neural network, image encryption, cybersecurity

## Abstract

The demand for cybersecurity is growing to safeguard information flow and enhance data privacy. This essay suggests a novel authenticated public key elliptic curve based on a deep convolutional neural network (APK-EC-DCNN) for cybersecurity image encryption application. The public key elliptic curve discrete logarithmic problem (EC-DLP) is used for elliptic curve Diffie–Hellman key exchange (EC-DHKE) in order to generate a shared session key, which is used as the chaotic system’s beginning conditions and control parameters. In addition, the authenticity and confidentiality can be archived based on ECC to share the EC parameters between two parties by using the EC-DHKE algorithm. Moreover, the 3D Quantum Chaotic Logistic Map (3D QCLM) has an extremely chaotic behavior of the bifurcation diagram and high Lyapunov exponent, which can be used in high-level security. In addition, in order to achieve the authentication property, the secure hash function uses the output sequence of the DCNN and the output sequence of the 3D QCLM in the proposed authenticated expansion diffusion matrix (AEDM). Finally, partial frequency domain encryption (PFDE) technique is achieved by using the discrete wavelet transform in order to satisfy the robustness and fast encryption process. Simulation results and security analysis demonstrate that the proposed encryption algorithm achieved the performance of the state-of-the-art techniques in terms of quality, security, and robustness against noise- and signal-processing attacks.

## 1. Introduction

The security of the data, infrastructure, and applications used to store, process, and transmit information falls under the name of cybersecurity, which is the method used to respond, monitor, and protect transmitted data and information. Through a cybersecurity perspective, the focus is on the secure exchange of information. However, current encryption methods are not reliable for effective image encryption [1]. To achieve the security requirements, we use the sensitivity property of the initial condition and control parameters inherent in chaotic methods. Based on the propagation and confusion properties of Shannon’s diffusion, the security of the image ciphering algorithm is estimated. Chaotic maps are classified into two types: one-dimensional chaotic maps and higher-dimensional chaotic maps. One-dimensional chaotic maps with a single variable spanning discrete time steps include logistic maps, tent maps, and sine maps [2,3]. It is important to note that the chaotic maps have numerous flaws, including their small chaotic ranges and ease of conversion to the periodic map. We also point out that one of the flaws is the lack of sufficient control parameters [4,5,6,7]. On the other hand, chaotic maps with higher dimensions contain at least two variables. Their characteristics include chaotic orbits that are difficult to predict and improved performance [8,9], but due to their difficulty in implementation and high computing costs, they are not processed in real time [10,11].

The authors introduced a new one-dimensional chaotic system to overcome the limitations of one-dimensional chaotic maps. Based on the modular one principle, this system encodes images to increase the number of control parameters [11,12]. Yicong et al. [13] suggested a cascade chaotic system (CCS) as a general one-dimensional chaotic framework and used any two 1-D chaotic maps as seed maps to produce a new non-linear chaotic system. As for Zhongyun et.al, proposed dynamic parameters to control a chaotic system (DPCCS). DPCCS is a straightforward structure that dynamically adjusts the chaotic map’s other parameters using the control map’s output (seed map). Both CCS and DPCCS have an easy-to-implement hardware architecture, extremely chaotic behavior, and a basic structure. However, many weaknesses, such as time delays and hardware implementation difficulties, appear clearly when more initial maps are serialized [14].

The message hash or message digest is the fixed output, while the hash function is the encoding mechanism that converts the variable-length input message [15]. Hash functions are used by the majority of authentication systems, digital signatures, data integrity safeguards, and random number generators. In addition, we may determine the hash value by utilizing a contemporary cryptographic hash function. Because it is challenging to detect two different messages with the same hash value within the hash function, hash functions are frequently employed in popular security protocols (such as Transport Layer Security or Internet Protocol Security) (collision resistance). This characteristic enables the widespread usage of hash functions in widely used security protocols like Transport Layer Security and IP Security.

The Secure Hash Standard (SHA) was first released by NIST [16] in 1993. In 1995, SHA-1, an updated version of the SHA algorithm, was introduced in an attempt to address some of the original flaws. The SHA-2 hashing algorithm was proposed in 2001 [17]. Because it takes into consideration larger digest messages, it is more resistant to potential attacks and may be used with larger data inputs (up to 2128 bits in the case of SHA512) (DM). The size of the operands, initialization vectors, and final DM varies for the SHA224, SHA256, SHA384, and SHA512 hashing algorithms, but the SHA-2 hashing algorithm is the same for all of them [18]. One cipher alternative that is built using chaotic maps is the hash functions. Inputs of hash functions are of random size and outputs are of fixed length (message summary or hash value) divided into keyed and non-key variants. Hash functions have an important role in data look-up tables and message integrity checks. Message authentication can be added through the keyed variant of the hash functions. Uniform distribution is the form of distribution that is used to distribute hash functions, which are one-way functions and highly sensitive to any change in inputs. This sensitivity leads to obtaining a completely different hash value when making any slight change to the inputs. As it happens, the same characteristics are shared by chaotic maps. Therefore, to draw on these characteristics, many researchers have integrated chaotic maps in the hash functions [19,20,21,22,23,24,25,26].

For many years, there has been a famous encryption technique known as cryptography (asymmetric cryptography) [27]. For small devices, computationally strong public-key encryption is often too expensive if cryptographic hardware is not used to accelerate it. Effective public-key cryptosystems are frequently created using elliptic curves. Elliptic curve cryptography (ECC) is a well-liked technology with several benefits, including tiny storage requirements, quick computations, and low-power requirements [28]. The most well-known technique that used ECC, and also provided data security, is the Menezes Vanstone Elliptic Curve Cryptosystem (MV-ECC) [29]. This technology was used and made more convenient for image encryption and security. It is widely used for secure communication, since the elliptical curve, which is based on encryption systems, has excellent encryption properties. Applications for encrypting images with an asymmetric key method were introduced in [30]. The work that is being offered introduces a novel mechanism that transforms the image pixels into a set of points on elliptical curves over a finite field, GF (*p*). The collected points are then subjected to elliptic curve (EC) operations, which improve the security of the encryption system and result in higher performance. EC over the finite field GF (*p*) is the foundation of an image encryption technique proposed in [31]. Using the public key to encrypt the obtained points, the mapping table’s index value ranges from 0 to 255 while accounting for the image’s pixels and pixel positions.

To our knowledge, the proposed method is among the earliest publications to employ public key elliptic curve cryptography incorporations with deep CNN to achieve the authentication properties and integrity. Moreover, instead of using the privet hey for both block and stream cipher for designing the privet key generator, this work presents a new research direction for using the proposed APK-EC-DCNN to automatically realize private and public key generator and satisfy the authentication properties with a high-security level. The key contributions of the proposed method are summarized as follow:

(i) The DCNN is used with the public key elliptic cryptosystem in order to generate authenticated public key.
(ii) A new Quantum Chaotic authenticated PK-EC Deep CNN generator is introduced.
(iii) A robust APK-EC-DCNN partial image encryption based on the frequency domain is suggested.

The rest of this paper is organized as follows: in Section 2, the preliminaries that contain the PK-Elliptic curve cryptosystem and 3D Quantum Chaotic Logistic Map are discussed. In Section 3, the proposed PK-ECC-DCNN partial image encryption is introduced and discussed in detail. The simulation results and security assessments are displayed in Section 4. The proposed study’s conclusion is then provided in Section 5.

## 2. Literature Review

### 2.1. Deep-Learning Networks

Since complex functions can be effectively expressed by using a multi-layer network structure, deep learning has emerged as a recent trend in scientific research and is used in a variety of applications (such as image processing, image classification [32,33], object detection [34,35], and image segmentation [36,37]). One of the subfields of deep learning is the Generative Adversarial Network (GAN) [38], which consists of the generator and the discriminator. The generator must keep track of the distribution of the sample data, while the discriminator must identify whether the input is real data or a manufactured sample. An alternative to the stream cypher generator is the deep-learning model, which has a large number of parameters, a complex network architecture, and a random training process.

In [39], a new algorithm based on a deep convolutional generated adversarial network (SCGANs) was introduced for chaotic block image encryption, and the Feistel network was improved. A new system combined with a deep and hyperchaotic convolutional adversarial network has also been proposed. The major goal was to deliver a key stream of random numbers that was more complex and unpredictable. A deep-learning-based streaming cypher generator and a stream cypher generator that produces the private key required to encrypt and decrypt medical images were also introduced by the researchers in [40]. Lately, work has been done to introduce the chaotic algorithm-based key generation and deep convolutional neural network-based picture encryption technique [41].

Recently, in the industry 5.0 technology, a new secure SIELNet encryption technique for color images has been introduced [42]. In order to encrypt color images in the first place, the authors provided a novel three-dimensional chaotic map, which produces cypher images that are related to the original images and this chaotic map has a good chaotic behavior. Second, the authors tackle the restricted super-resolution problem by reconstructing a lossy image from a compressed, encrypted image using a tailored residual dense spatial network.

### 2.2. PK–Elliptic Curve Cryptosystem

In image encryption algorithms, there are two commonly used cryptosystems: stream cipher and block cipher. Stream ciphers generally have a higher level of security than block ciphers like the Advanced Encryption Standard, International Data Encryption Algorithm, and Data Encryption Standard (AES), and they are also faster in terms of encryption and decryption speeds. They have low error propagation and better synchronization, and are cheaper to implement [43,44]. Linear feedback shift registers [45], nonlinear feedback shift registers [46], finite automation [47], linear congruence generators [48], and chaotic systems [49] are popular stream cypher generators. Public key encryption is more robust than the stream and block cipher. The elliptic curve discrete logarithm problem is the best algorithm now available for tackling the underlying mathematical problem of ECC, and it requires full exponential time. On the other hand, RSA and DSA rely on subexponentially-temporal techniques to solve the discrete logarithm and integer factorization problems [50]. This suggests that, compared to methods for integer factorization and discrete logarithm issues, techniques for addressing the elliptic curve discrete logarithm problem become infeasible considerably more quickly as the problem size increases. Due to the much-reduced key size, ECC provides security levels comparable to RSA and DSA [50].

In this section, an overview of the ECC in data security was presented in [51]. In [52], the concept of the Kobltiz technology, which encodes and decodes visual data to the points of an elliptical curve, was introduced. The approach in [53] alters the dimensions of the input images first by using the k parameter. In [54], a matrix-based image coding strategy was presented using a fast-mapping technique. Alphanumeric character values are converted into (*x*, *y*) coordinates for elliptic curves utilizing various matrix characteristics, an elliptical curve, and a non-singular matrix. The mapping technology employed in the plan ensures the confidentiality of the encrypted data, enhancing the encryption system’s security. ECC and its use in data security are surveyed by the authors in [54]. The authors compare the ECC-based encryption scheme to the current asymmetric key ciphering protocol and examine the ramifications of each. The ECC scheme’s security weaknesses are also described.

The author compared the ECC-based encryption system to other asymmetric key cryptography methods in use today, such as RSA, and evaluated its drawbacks. The authors demonstrated how the ECC system has security issues. The majority of systems have limited key spaces and lengthy execution times. With the cubic equation, an elliptic curve EC over a finite field *Fp* is defined [51]:

(1) $$y^{2}=x^{3}+ax+b\;mod\;p$$

where p is a large prime number and a, b are two integers which are smaller than p and satisfies the following condition:

(2) $$4a^{2}+27b\;mod\;p\neq \;0$$

In the above equations, each value of a, b ϵ p provides a different elliptic curve $EC_{p,a,b}$ where a, b, and p are the parameters of the elliptic curve. The PK-EC Diffie–Hellman Key Exchange [40] can be discussed as: Le G is a base point of an EC, $P_{A}$ and $\;P_{B\;}$ can be computed as [39]:

(3) $$P_{A}=n_{A}\cdot G,\;P_{B}=n_{B}\cdot G$$

where $P_{A}$, $\;P_{B\;}\;$ are the sender’s and receiver’s public keys, and $n_{A}$, $n_{B}$ are the respective private keys. The transmitter and receiver calculate the shared key as $n_{A}P_{B}$ and $n_{B}P_{A}$, respectively.

(4) $$Sk=n_{A}\;P_{B}=n_{A}n_{B}G=n_{B}n_{A}G=n_{B}P_{A}\;$$

For a specific point G on the EC of elliptic curve $EC_{p}\;\left(a,\;b\right),$ the private key n is exponentially complex to solve, where $P_{u}$ = nG, *G* is a base point, while n is a random integer. It is impossible to determine the discrete logarithm of $P_{u}$ with respect to a widely known base *G*. The elliptic curve discrete logarithmic problem (ECDLP) is significantly more secure than other encryption techniques that rely on the integer factorization problem for a certain key size [51].

### 2.3. 3D Quantum Chaotic Logistic Map

An excellent example of a complex chaotic map that results from nonlinear dynamical equations is the quantum logistic map, which is generated by the classical logistic system and is a quantized version of a classical chaotic system [55,56,57,58]. While chaotic maps in higher dimensions, such as the one used in the proposed scheme, can result in an expansion of the key space range, they are overly complex, highly random, and highly sensitive to initial conditions and control parameters, as opposed to classical chaotic maps, which have low control parameters and, as a result, a small chaotic range. Therefore, using the quantum logistic system as the seed system in an encryption method is appropriate [59]. A quantum chaotic map was presented in [55], which is defined as:

(5) $$x_{n+1}=r\left(x_{n}-{\left|x_{n}\right|}^{2}\right)-ry_{n}$$

(6) $$y_{n+1}=-y_{n}e^{-2\beta }+e^{-\beta }r\left[\left(2-x_{n}\right)y_{n}-2x_{n}z_{n}\right]$$

(7) $$sz_{n+1}=-z_{n}e^{-2\beta }+e^{-\beta }r\left[\left(2(1-x_{n}\right)z_{n}-2x_{n}y_{n}-x_{n}\right]$$

**Figure 1:**
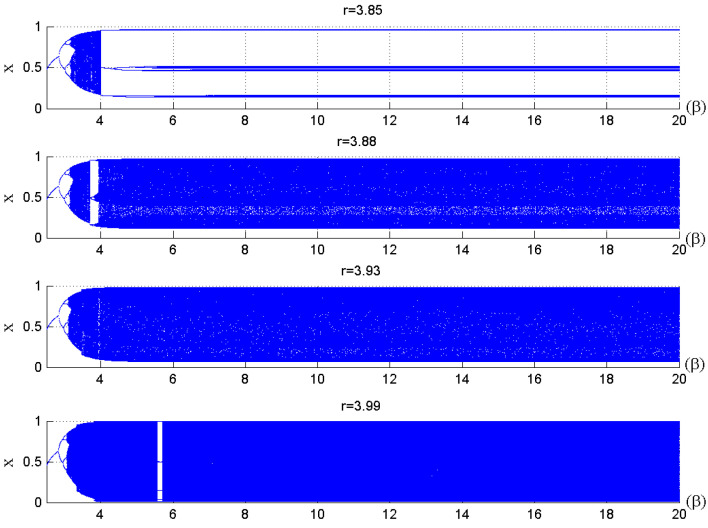
Quantum map bifurcation diagram for various control parameters (*r*) [55].

**Figure 2:**
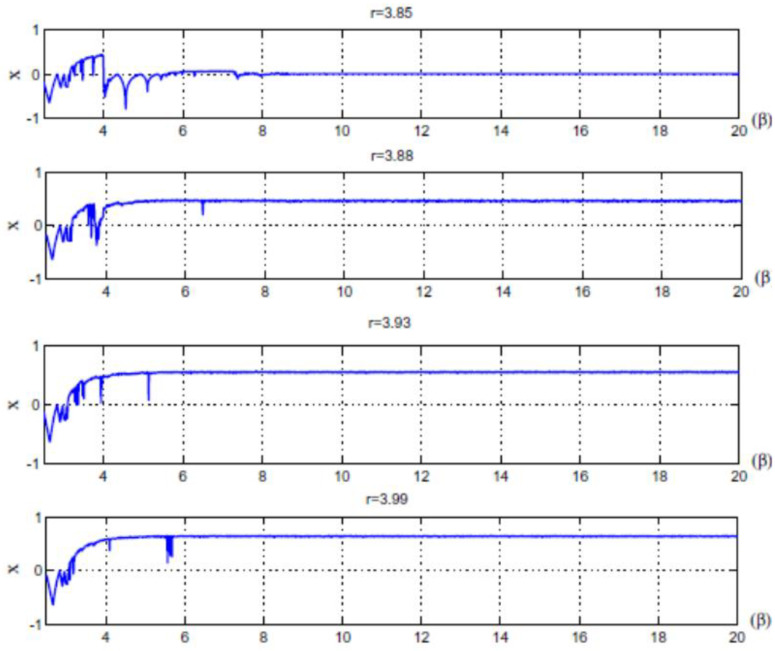
Quantum map’s Lyapunov exponent for various control parameters (*r*) [55].

The main parameters used in the quantum chaotic map are the control parameter, the parameter, and the $x_{0},y_{0},z_{0}\;$. respectively. The advantages of the quantum logistic map are its huge capacity, natural parallelism, and straightforward structure. Additionally, the chaotic patterns produced are more pseudorandom. For various control parameters, the quantum map’s bifurcation diagram and Lyapunov exponent are provided in [55]. When *r* ≥ 3.99 and *β* ≥ 6, the quantum chaotic map can exhibit better chaotic behavior, as shown in Figure 1 and Figure 2.

## 3. Proposed PK-ECC-DCNN Cryptosystem

In this section, the proposed PK-ECC-DCNN cryptosystem and its application in image encryption and authentication will be discussed. In the proposed image encryption cryptosystem, the PK-ECC Diffie–Hellman Key Exchange is used as a key exchange model based on the difficulty of the discrete logarithmic problem. The secret 3D quantum chaotic logistic map initial condition values and the control parameters will be extracted from the PK-ECC key exchange, which stuffy the authentication property. Based on the PK-ECC key exchange and the authentication property, the proposed authenticated chaotic DCNN key generator design is investigated. The outputs of the 3D quantum chaotic logistic map and the secure hash function are used to produce the proposed authenticated expansion diffusion matrix and the permutation confusion matrix. In the proposed cryptosystem, the partial image encryption can be achieved by using the discrete wavelet frequency domain transform.

### 3.1. Public Key Deep CNN Generator (PK-DCNNG)

**Figure 3:**
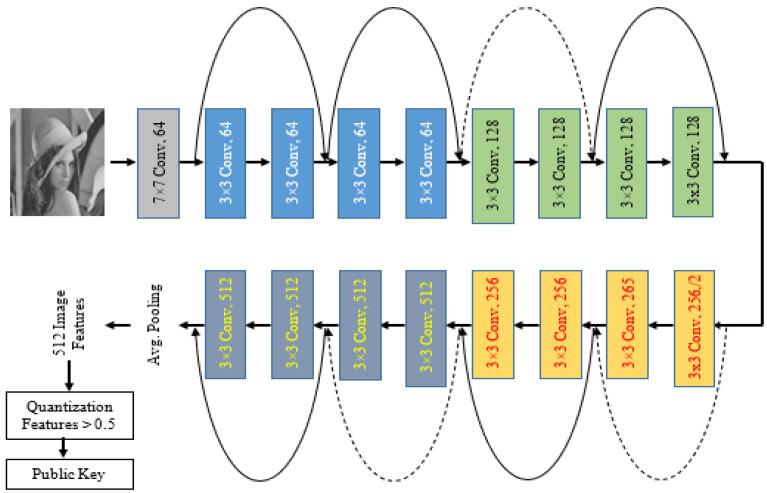
Proposed ResNet-18 CNN for Public Key Generation.

In this section a public key is generated using Convolutional Neural Networks (CNNs). The pre-trained ResNet-18 convolutional neural network was used. This network is 18 layers deep [60], and it has been trained on more than 1-million images from the ImageNet database. This pre-trained network can categorize images into 1000 object categories, such as pencil, mouse, keyboard, and many more animal images. Thus, the network got acquainted with a lot of features for a large group of images. It is often much easier and faster to use transfer learning via pre-trained networks than to train a designed convolutional network from scratch. The proposed network model architecture is illustrated in Figure 3. The network contains 18 layers, 3 × 3 filters are used in convolutional layers, and the network is set up to ensure the number of filters is the same as the number of layers, if the output feature map is of the same size. However, when the output feature map is split in half, the filters are doubled in layers. By means of the convolutional layers by stride of 2, the down sampling is performed. The residual shortcut connections are inserted between layers throughout the network [61]. There are two types of connections. The first type is indicated by solid lines and is used when the input and output dimensions are the same. While the other type is used when increasing the dimensions, this type is referred to as the dotted lines. Identity mapping has been performed by this type of connection but with zeros padding with a stride of 2 for increased dimensions.

The input photos are presented hierarchically by the network. Higher-level features that were acquired utilizing the lower-level features of the preceding layers are found in deeper layers. Through the use of activations on the global pooling layer, also known as “pool5,” we can acquire feature representations of training and test data at the conclusion of the network. In total, we obtain 512 features by aggregating the input features throughout all spatial areas by means of the global pooling layer. Through these image features, we obtain a value between 0 and 1. Finally, these values are converted to binary by comparing them with 0.5. If these values are less than 0.5, they will be 0. Otherwise, they will be 1.

### 3.2. Authenticated PK-Chaotic DCNN Key Generator

**Figure 4:**
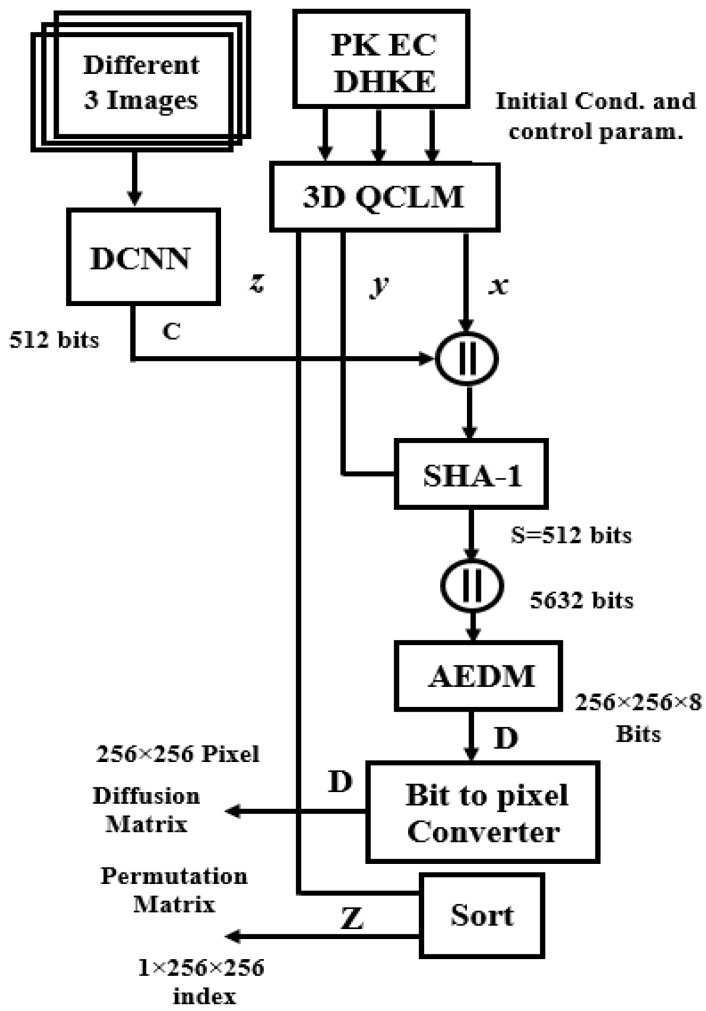
Proposed Authenticated chaotic DCNN Key Generator (AC-DCNN-KG).

**Figure 5:**
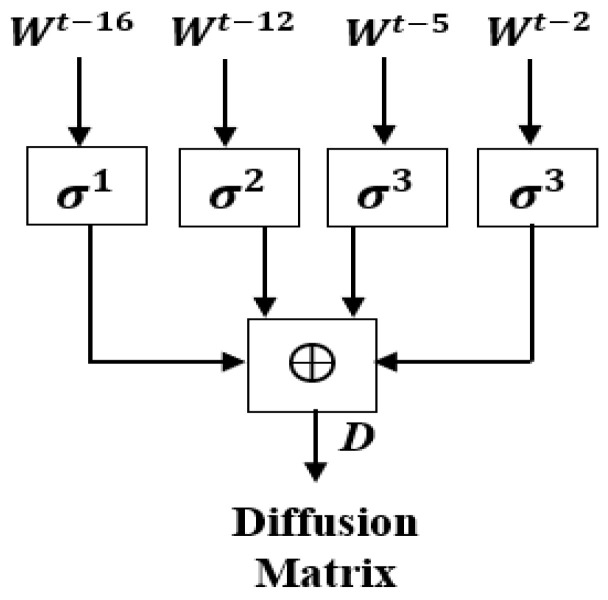
The proposed Authenticated Expansion Diffusion Matrix (AECM).

In this section, authenticated chaotic DCNN Key Generator (APK-C-DCNN-KG) has been investigated. Figure 4 and Figure 5 show a block diagram of the proposed chaotic DCNN-AKG. The proposed structure chaotic DCNN-AKG consists of the proposed chaotic map, the DCNN, secure hash function (SHA-1) with output length 8 × 32-bit words, and the authentication expansion diffusion Matrix (AEDM). The SHA-1 has two concatenated inputs. The first input is the output of the DCNN, and the second is the variable chaotic secret values and lengths obtained from the proposed chaotic map, which can be vary from 128 × 32-bit words to 512 × 32-bit words. The SHA-1 is used for key authentication for both DCNN output and the variable chaotic secret values and lengths. The AEDM inputs are the 8 32-bit words output of the SHA-1 and second output of the proposed chaotic map of length varies from 64 32-bit words to 256 × 32-bit words. Figure 4 depicts the proposed AEDM’s block diagram and the final output of the proposed AEDM is 2048 × 32-bit word. The proposed AEDM ultimate result can be converted to 256 × 256 × 8 bits, and then converted to 256 × 256 pixel as the size of the original image for the diffusion encryption step. The first and second variable chaotic secret lengths from the proposed chaotic map, which can be used as an input to the SHA-1 and the proposed AEDM, are provided from the PK-EC Diffie–Hellman Key Exchange and the proposed 3D QCLM. The expanded diffusion values $W^{17}$ to $W^{L}$ are provided by:

(8) $$W^{L}=\sigma^{1}(W^{t-16\;})+\sigma^{2}(W^{t-12\;})+\sigma^{3}(W^{t-5})+\sigma^{4}(W^{t-2\;})$$

where “*t*” is the index parameter started from 17 to L; $\sigma^{1}$, $\sigma^{2}$, $\sigma^{3}$, $\sigma^{4}$ are provided by:

(9) $$\sigma^{1}\left(•\right)=R^{8}\left(•\right)⊕R^{12}\left(•\right)⊕R^{20}\left(•\right)$$

(10) $$\sigma^{2}\left(•\right)=R^{3}\left(•\right)⊕R^{10}\left(•\right)⊕R^{17}\left(•\right)$$

(11) $$\sigma^{3}\left(•\right)=R^{5}\left(•\right)⊕R^{19}\left(•\right)⊕R^{22}\left(•\right)$$

(12) $$\sigma^{4}\left(•\right)=R^{2}\left(•\right)⊕R^{16}\left(•\right)⊕R^{26}\left(•\right)$$

In addition, the operations “+” is mod $\;2^{32}$ addition, $“R^{n}”$ is right shift by “*n*” bits, and “⊕” is the XOR operation.

The block diagram of the proposed authenticated public key generation is shown in Figure 4. Figure 4 shows that the proposed authenticated public key generator depends on the proposed deep convolutional neural network and the proposed authentication expansion diffusion Matrix in order to achieve the authentication strategy. The proposed Authenticated Expansion Diffusion Matrix (AECM) design is shown in Figure 5.

### 3.3. Proposed APK-EC-DCNN Partial Image Encryption

**Figure 6:**
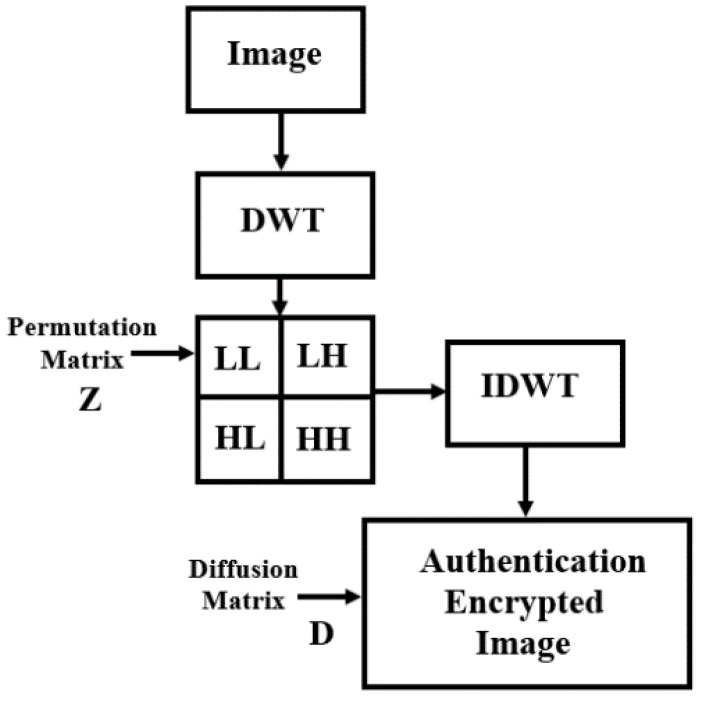
The block diagram of the proposed authenticated encryption algorithm.

This study proposes a new scheme for partial image encryption. A new authenticated public key deep convolutional neural network was proposed in order to authenticate the public key generated by the Deep Convolutional Neural Network. In addition, the encrypted key exchanged scheme is based on the public key elliptic curve Diffie–Hellman key exchange protocol. This encrypted key exchange depends on how challenging the discrete logarithmic problem is to solve. The block diagram of Proposed PK-EC-APK-DCNN partial image encryption/decryption algorithm is shown in Figure 6.

**Figure 7:**
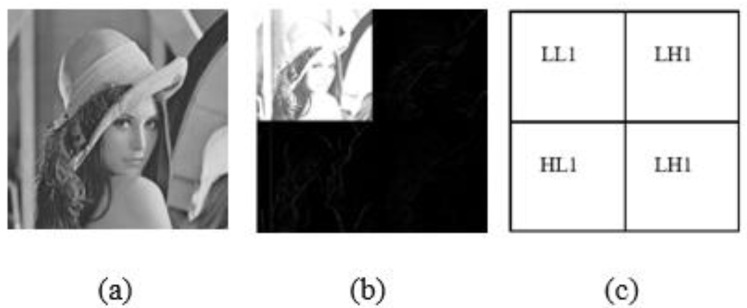
DWT: (**a**) Lena image (**b**) schematic diagram of the two-layer wavelet decomposition of Lena; (**c**) wavelet decomposition sub-bands.

In addition, in order to achieve real-time processing, the partial encryption technique based on the discrete wavelet transforms where only a quarter part of all images will be encrypted so it can save computational time and cost. The two-dimensional (2D DWT) is used to achieve real-time processing. Each level of decomposition in 2D DWT yields four bands of data, one of which corresponds to the low pass band (LL), and the other three to the high pass bands (horizontal (HL), vertical (LH), and diagonal (HH)). Figure 7 shows the one-level wavelet decomposition of Lena and its corresponding wavelet decomposition sub-band mod. As illustrated in Figure 7, the 2-D DWT can be built utilizing two digital channel filters and a collection of down samplers. A Low-Pass Filter (LPF) and a High-Pass Filter (HPF) are the digital filters that are utilized. The image is subjected to a first-level (1-L) 2-D DWT to produce four distinct sub-images. In addition, in this paper, we used the Haar filter, which is a popular filter in the wavelet family.

Authenticated Key Generation, confusion, and diffusion process are three steps used in the suggested partial encryption scheme. The Authenticated Key Generation is discussed in Section 3.2 in Algorithm 1. The confusion process is performed based on the third output of the 3D QCLM, in which a high degree of scrambling and randomization can be achieved. In addition, the diffusion process is used to change the image pixel values for cryptographic process. The authentication property can be achieved as follows:

1. Authenticated public key elliptic curve based on deep convolutional neural network has been introduced in the paper.
2. The public key elliptic curve cryptography incorporates with deep CNN is used to achieve authentication properties and integrity.
3. Authenticated expansion diffusion matrix (AEDM) is designed in this paper based on the secure hash function (SHA), which uses the output sequence of the DCNN and the output sequence of the robust chaotic map in order to satisfy the authentication property.

**Algorithm 1.** PK-Authenticated Key Generation1.1 Deep CNN step Step 1Read the multi-image matrixes with the same size in Concatenated Structure A, BStep 2Use the multi-image matrixes with the same size in concatenated structure as input to the DCNN to generate 512 output bits (C)Step 3Extract the initial conditions and the control parameters from the PK-EC Diffie–Hellman Key Exchange (PK EC DHKE) for the 3D QCLMStep 4Run the 3D QCLM and concatenate the 5120 bits from the ‘x’ (the first dimension) output with the output of DCNN 512 bits to get the 5632 bits (*L*)1.2 SHA-1 and AEDM Step 5Use the 5632 bits (*L*) as input to the SHA-1 to produce authenticated 256 bits (S)Step 6Run the 3D QCLM and concatenate the 5120 bits from the ‘y’ (the second dimension) output and concatenated with the authenticated 256 bits (S)Step 7The AEDM algorithm used the concatenated $\left(y\|S\right)$ bit vector of 5632-bit length in order to produce the diffusion matrix “D” of length 256 × 256 × 8-bit vector by using Equations (12)–(16)Step 8Convert the diffusion matrix “D” to 256 × 256 pixel (the original image size)Step 9The chaotic sequences of the third dimension “z” output of the 3D QCLM output is obtained after a predetermined number of iterations is used to generate the permutation index matrix of length 128 × 128 sorted values based on the “z” output of the 3D QCLM as:
*Z* = sort (*z*)                                                                                                                                                                                                       (13)
Here, the function sort (.) returns a random index vector with the ascending order

#### 3.3.1. Encryption Process

***Step 1****:* Applying DWT to the image “I” yields the approximation matrices LL, diagonal HH, horizontal HL, and vertical LH.

***Step 2****:* The permutation step is done in the frequency domain (the approximation LL’ matrix), by using the sort function indicated in Equation (13); this will cut down on calculation time and cost.

***Step 3****:* Apply IDWT for all sub-bands (LL’, HH, HL, LH) to reconstruct the permuted images.

***Step 4****:* Based on the authenticated diffusion matrix, the diffusion process can be achieved by using the exoring operation between the output of the IDWT step with the output of the authenticated encrypted diffusion matrix (D).

***Step 5****:* The second user uses the same keys and secret same parameters to generate the authenticated encryption diffusion matrix and the permutation index matrix.

#### 3.3.2. Decryption Process

***Step 1****:* Based on the PK-EC Diffie–Hellman Key Exchange, the initial conditions and control parameters for the 3D QCLM can be generated.

***Step 2****:* The second generates the same keys and the secret same parameters to generate the authenticated encryption diffusion matrix and the permutation index matrix.

***Step 3****:* The first step in the decryption process is exoring operation the encrypted image with the output of the authenticated encrypted diffusion matrix (D).

***Step 4****:* The second step in the decryption process started by applying DWT to the encrypted image yields the approximation matrices “LL,” diagonal “HH,” horizontal “HL,” and vertical “LH.”

***Step 5****:* Applying the de-permutation on the (sub-band) approximation matrices “LL” to obtain the de-permutation sub-band “LL.’’

***Step 6****:* Applying the IDWT to the all-sub-bands (“LL,’’ “HH,” “HL,” “LH”) to generate the decrypted image.

## 4. Simulation Results and Security Analysis

The parameters selected for EC in 64-bit in this study are *p* = 113, *a* = −1, *b* = 17, and *G* = (52,61). The proposed method will be used for grayscale images and Lena images for results comparison. By measuring the resistance of a cipher system to various attacks (such as statistical attack, known plaintext attack, differential attack, various brute force attack, and ciphertext attack), the robustness of the cipher system is evaluated. In order to evaluate the proposed scheme, a safety analysis was conducted on it by discussing entropy, histograms, correlation coefficient NPCR, UACI, MSE, and the NIST and randomness tests.

### 4.1. Randomness Test

The proposed AC-DCNN-KG randomization was tested using the 16 statistical tests that made up the NIST test suite. By means of these tests, it is possible to determine whether or not the generated sequence is random. Among these tests, the probability value (the *p*-value) is mainly relied upon. The boundary between the region of rejection or non-rejection is the comparison of the *p*-value to the level of significance. In Nest, the significant level is 0.01. When the value of P is less than 0.01, it means that the order is not arbitrary and, therefore, rejected. The order is reasonable and arbitrary when the *p* value is bigger than 0.01, though. The proposed authentication expansion diffusion matrix’s binary sequence of $10^{6}$ bits is tested using SP800-22 [62,63], and the results are shown in Table 1.

### 4.2. Chi-Squared (Histogram) Analysis

**Figure 8:**
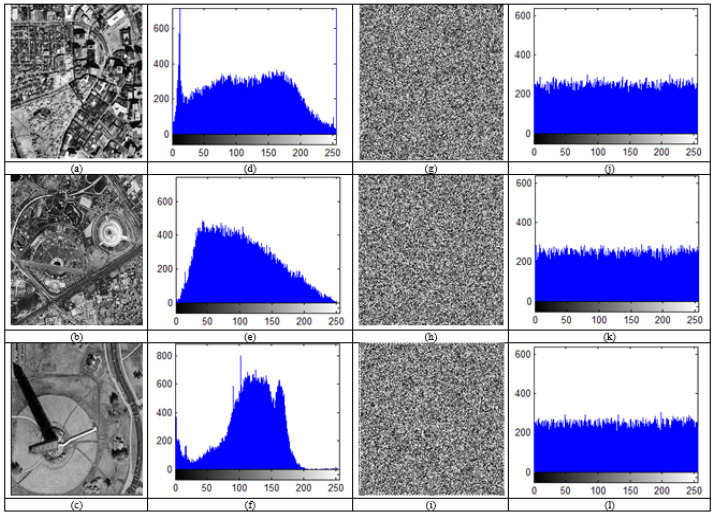
Histogram of satellite images: (**a**–**c**), sat1; sat2; sat3 images; (**d**–**f**) Histogram of (**a**,**c**,**d**); (**g**–**i**) encrypted images of (**a**–**c**); (**j**–**l**) histograms of (**g**–**i**).

We can spread the image’s pixel intensities by plotting the histogram. For effective encryption, the histogram of the encrypted image must be flat and homogeneous. As a result, there is no connection between the original image and the encrypted image, and no data that could be used to decode the original image. Figure 8 shows the histogram of satellite images sat1, sat2, and sat3. In addition, Figure 8d–f indicates the histogram of (a–c); (g–i) encrypted images of (a–c); (j–l) histograms of (g–i). Finally, as seen in Figure 8, the histogram of the encrypted image is regular and notably different from the histogram of the original image as a result of the diffusion and confusion used in the suggested approach. Thus, it is protected from statistical assaults.

The chi-squared test ($\chi^{2}$), created by renowned statistician Pearson is used to compare two frequencies: the expected frequency and the observed frequency from the experiment [64]. This test is crucial in determining how resilient an encryption is against statistical assault in image encryption. The histogram of an image’s distinctive pattern should be able to be changed by the encryption method into a straight line-like no-pattern known as a uniform distributed histogram. In a conventional image, each grey level has a different frequency, whereas in an encrypted image, each grey level must have the same frequency. Using Equation (14), the Chi-squared test calculated the difference in frequency between the plain image and the encrypted image [64].

(14) $$\chi^{2}=\sum_{k=1}^{256}{\left(U_{k}-M\times N/256\right)}^{2}$$

In the above-mentioned Equation (14), where M×N/256  is the predicted frequency and $U_{k}$ represents the observed frequency of each grey level (0–255) for an original image, the difference between the two frequencies must be small in order for the test to be considered successful. When the Chi-square value is equal to α = 0.05, the critical Chi-square value $\chi_{0.05}^{2}\left(255\right)$ = 293.2478.

In order to use the $\chi^{2}$ test, we prepere differents encrypted images by changing 1-bit of a pixel at a time different in original images. The Chi-square value score of different encrypted images is listed in Table 2, which proves that the proposed cryptosystem uniformly distributed the pixel values from 0 to 255. In Table 3, the Chi-square results test for 100 encrypted Lena images under α = 0:05 is studied and compared with recent researches.

### 4.3. Correlation Coefficient

**Figure 9:**
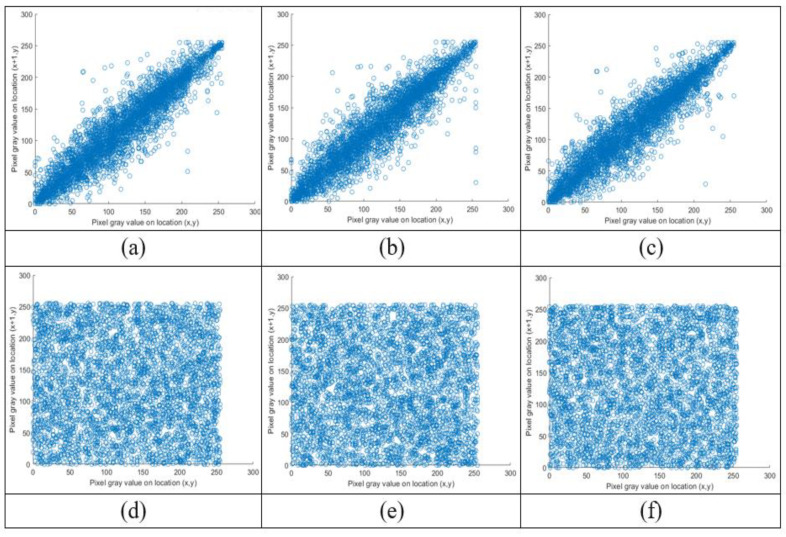
Lena correlation analysis of ciphered image in three directions. (**a**,**b**) Horizontal correlation of the plain image and ciphered image. (**c**,**d**) Vertical correlation of the plain image and ciphered image. (**e**,**f**) Diagonal correlation of the plain image and ciphered image.

Each pixel in the original image has a strong correlation with its surrounding pixels. There is no correlation between neighboring pixels in the vertical, horizontal, or diagonal directions thanks to the strong encrypted system, which minimizes this correlation to the lowest feasible value. The correlation between neighboring pixels of the satellite image is shown in Figure 9a–c, and its cypher image correlation is also obtained in Figure 9d–f in the horizontal, vertical, and diagonal directions. In an image, the correlation coefficient $C_{r}$ from Equation (15) is calculated between each pair of neighboring pixels. The correlation value should be close to zero [41]. The estimated correlation coefficients for each plain image and its associated cypher image are compared in Table 4 for each image.

(15) $$C_{r}=\frac{\sum_{i=1}^{N}(x_{i}-E\left(x\right))(y_{i}-E\left(y\right))}{\sqrt{\sum_{i=1}^{N}(x_{i}-E\left(x\right))\sqrt{\sum_{i=1}^{N}{\left(yi-E\left(y\right)\right)}^{2}}}}\;$$

The correlation coefficients of the encrypted images, which were close to zero while the correlation coefficients of the original images were close to one, clearly show that the proposed technique is very robust to statistical attacks. The $C_{r}$ values of the ciphered images are very near to zero. As a result, the suggested encryption system has good confusion and diffusion properties and can withstand statistical attacks.

### 4.4. Information Entropy

Entropy, which indicates uncertainty in the cypher image, is used to calculate the unpredictability of the received image. Strong randomness and confidentiality are indicators of a high entropy for the encoded image [65,66,67]. According to one definition, the information system’s entropy is specified as:

(16) $$H\left(m\right)=-\sum_{i=0}^{2^{N}-1}p\left(m_{i}\right)log_{2}p\left(m_{i}\right)$$

where “m” is the source of information, N total number of bits represents the symbol $\;m_{i}$, $p_{\left(m_{i}\right)}$ probability of symbol $\;m_{i}$, the best value of the information entropy close to the value 8.

According to Table 5, which shows the information entropy of the ciphered image created by our algorithm to be close to 8, the suggested cryptosystem has a low probability that an attacker will be able to decode a cypher image.

### 4.5. Key Space Analysis

The collection of all keys used in an image encryption system is known as key space. This key set should be sufficiently big to fend off a brute force attack and produce a successful strategy. This scheme can be evaluated by measuring the sensitivity of the key and the number of keys. In the proposed scheme, assuming that the double-precision binary floating-point IEEE 754 format is employed as $\;10^{-15}$, the 3D QCLM has different five secret parameters ($x_{0},y_{0},z_{0},\;r,\;\beta$) with $\;10^{16\times 5}$ = $10^{80}$. The SHA output is 256 bits, so it has a key space of $\;2^{256}$. In addition, the keys will be exchanged by means of a 256-bit elliptic curve parameter using ECC. The raw parameters of the logistic map are derived to generate a 256-bit sequence using secretly shared elliptic curve point nG. The recipient receives the shared key nG as encrypted data $P_{u}$. It is extremely hard to extract nG from $P_{u}$, which necessitates knowing the private key n. Note that ECC has a little key size if compared with RSA [30]. In addition, the 256-bit key is used to generate the key matrix. The total key space of the proposed cryptosystem is $2^{924}$. It is known that the minimum key space necessary to resist brute-force attacks is $\;2^{100}$, so the entire key space for the proposed cryptosystem is very large. Table 6 provides a comparison of key space with current designs.

### 4.6. Key Sensitivity Analysis

**Figure 10:**
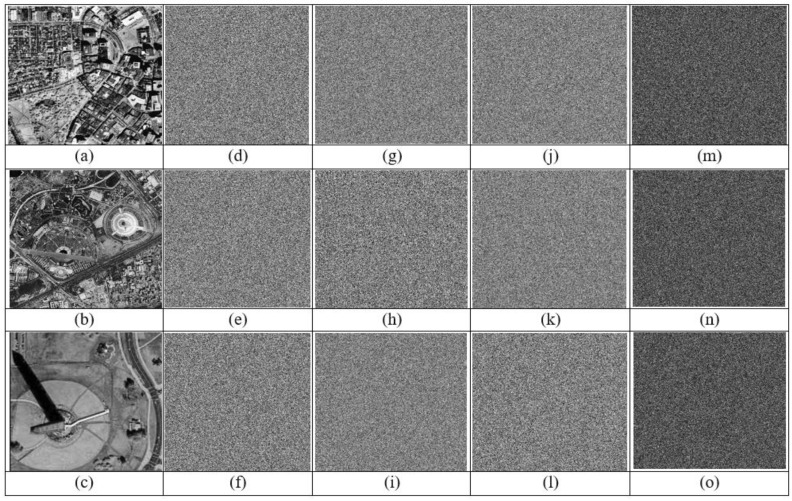
The key sensitivity analysis for the three different selallite images. (**a**–**c**), sat1; sat2; sat3 are the original images; (**d**–**f**), (**g**–**i**), (**j**–**l**) the encrypted three images with modified different 3-bit key in each image. (**m**–**o**), illustrate the differences between the encrypted images.

A robust encryption technique has high-key sensitivity across the board. As a result, a slight modification to the key results in the production of a completely different cypher image. Additionally, a small modification to the decryption key prevents the recovery of the original sound [28]. By encrypting the various images with the proper key as shown in Figure 10, the suggested approach tests the sensitivity of the key in different three satellite images (sat1, sat2, sat3) (a, b, c). The encrypted image in Figure 10g–l is decrypted using a working different key with a very small changes in the correct key. A slightly different key than the one used to encrypt the original image is used in Figure 10m–o. These significant discrepancies demonstrate that the suggested approach has a high raw key sensitivity and, as a result, a robust resistance against statistical attacks.

### 4.7. Differential Attack Analysis

Diffusion performance is one metric used to gauge an image encryption system’s strength. That implies that the plain image’s pixels have a significant influence on the cypher image’s pixels. This time, the resistance to the differential attack can be assessed by altering the plain image by one bit and comparing the variations in the cypher images; as a result, a whole different cypher image must be provided. The image coding scheme is secured using two quantitative measures against differential attack, the two measures are the average intensity of uniform change and the rate of pixel change (*NPCR*) [66].

(17) $$NPCR=\frac{\sum_{i,j}D\left(i,j\right)}{M\times N}\times 100\%\;$$

(18) $$UACI=\frac{1}{M\times N}\frac{\sum_{i,j}\left|C_{1}\left(i,j\right)-C_{2}\left(i,j\right)\right|}{255}\times 100\%\;$$

where *M* and *N* are the width and height of the plain image, respectively, and *C*_1_(*i, j*) and *C_2_*(*i, j*) are the values of the pixels in the position (*i, j*) of the two cypher pictures. The number of grey levels is 255; *C*_1_ and *C*_2_ are the plain image before and after changing one bit; and *D* (*i, j*) is specified as follows:

(19) $$D\left(i,j\right)=\left\{\begin{array}{c} 0,\;for\;C_{1}\;\left(i,\;j\right)=C_{2}\;\left(i,\;j\right)\;\\ 1,\;for\;C_{1}\;\left(i,\;j\right)\neq \;C_{2}\;\left(i,\;j\right) \end{array}\right.\;$$

The conceptual values of 𝑁𝑃𝐶𝑅 = 99.61% and 𝑈𝐴𝐶𝐼 = 33.46. When the *NPCR* and *UACI* values are greater than or equal to these theoretical values, a better and more secure coding system is obtained. The value of one randomly chosen pixel was changed for the suggested technique, and the cypher images of the original image and the modified image, *C*_1_ and *C*_2_, were then calculated respectively, and *NPCR* and *UACI* were calculated for the different images. The result is shown in Table 7.

### 4.8. Noise Attack Analysis

A data loss attack occurs when a potential intruder intentionally removes pixels from a specific area of an encrypted image or loses some of the image as it is being transmitted over the internet. If a cryptosystem recovers the crucial data from the encrypted image, which loses the data, it is flawless. Images often contain information, but during transmission, some noise contaminates the image [65,66]. The original information in the image can be missed if noise appears. (e.g., the sound of pepper and salt). This article examines the impact of our encryption system on the image of Lena, and different salt and pepper noise ratios serve as indicators of the intensity of the attack. Gaussian noise, salt and pepper, speckle noise, and the filtering attack are studied for the noise attack analysis [65,66]. In this section, we added the ciphertext image with the noise before decryption step. The PSNR is used to evaluate the degree to which the proposed technique obscures the image encrypted in order to show its efficacy. The lowest possible PSNR value is ideal for the highest level of encryption. Using the following mathematical formula, PSNR is determined [65,66]:

(20) $$\mathrm{PSNR}=10log_{10}\frac{M*N*{\left(2^{n}-1\right)}^{2}}{\sum_{i=1}^{N}\sum_{J=1}^{M}{\left[I\left(i,j\right)-C\left(i,j\right)\right]}^{2}}$$

where *I* (*i*, *j*) is the pixel value in plane image at pixel point (*i*, *j*), and *C* (*i*, *j*) is the pixel value in cipher image at pixel point (*i*, *j*). Figure 10 displays the decrypted image with various noise levels. Table 8 shows the PSNR for different encrypted images, and it concluded that the PSNR possesses a very small value, which means that the proposed cryptosystem has a robust encryption algorithm.

Table 9 and Table 10 show the Gaussian noise, salt and pepper, speckle noise, and filtering attack effects on the decrypted Lena images and its PSNR values. These tables demonstrate that the proposed technique can withstand noise attacks by showing that when the noise reaches different level values, the decryption result can also be detected with the human eye.

### 4.9. Computational Complexty

The relationship between complexity and cryptography is very important in encryption algorithm designs. The encryption algorithms consist of the key generation step and the image encryption step. In the key generation step, the time complexity of generation chaotic sequence with M×N length is $O\left(M\times N\right)$. In addition, the image encryption step, pixel permutation, and diffusion are both linear operations in which the time complexity of permutation is $O\left(M\times N\right)$ and the time complexity of diffusion is the time complexity of permutation. Finally, the total time complexity of the image encryption step is $O\left(2\times M\times N\right)$ [67,68].

In the proposed cryptosystem, using DWT in the image encryption, only the approximation matrix LL is permuted in the frequency domain. Therefore, the time complexity of permutation can be obtained by $O\left(M\times N/4\right)$. The diffusion process is applied after using the IDWT for to the four sub-bands (LL, HH, HL, LH). Then, the time complexity of the encryption process is $O\left(M\times N\right)$. The total time complexity of the proposed cryptosystem can be computed as $O\left(M\times N/4\right)$ + $O\left(M\times N\right)$. Finally, the proposed partial image cryptosystem is faster than the fully image cryptosystem. At an image with the size of M×N (M = 256, N = 256), the time complexity of the full encryption process is equal to $O\left(2\times M\times N\right)$ = 131,072. For the proposed partial encryption, the time complexity is equal to $O\left(M\times N/4\right)$ + $O\left(M\times N\right)$. Therefore, the time complexity is 81920, and computational complexity saving is 37.5% compared with any full encryption scheme. For images with a size of M×N where M×N are (512 ×512) and (1024 ×1024), the computational complexity saving will be increased.

## 5. Conclusions

A new elliptic curve with public key authentication based on deep convolutional neural network (APK-EC-DCNN) for cybersecurity image encryption application is suggested. The public key elliptic curve cryptography, incorporated with deep CNN, is used to achieve the authentication properties and integrity. In addition, an authenticated expansion diffusion matrix (AEDM) is designed in this paper based on the secure hash function, which uses the output sequence of the DCNN and the output sequence of the robust chaotic map in order to satisfy the authentication property. The experimental analysis shows that the proposed APK-EC-DCNN cryptosystem satisfies a very competitive encryption performance and can resist common attacks. In addition, the results indicate that the proposed cryptosystem has large key space, pseudo-randomness, one-time pad, and highly sensitive to change. In addition, the deep-learning mechanism is used in the encryption process, which considered a new direction of the encryption cryptosystems. The robustness of the encryption system is assessed by testing the resistance of the proposed system to numerous attacks. A safety analysis was done on the suggested method to evaluate it, and it included discussions of the histogram, entropy, correlation coefficient, NPCR, UACI, and NIST randomness tests.

This work contributes to the existing literature by further exploring the weaknesses of using the deep CNN as a feature extraction of an images as a secret key, which is used in recent works on image encryption. Moreover, the proposed cryptosystem introduces the use of public key elliptic curve cryptosystem in order to generate authenticated public key. In addition, an authenticated chaotic DCNN key generator is designed in order to achieved the authentication property. Moreover, the partial image encryption based on the frequency domain is used the discrete wavelet transform. The PK-EC Diffie–Hellman Key Exchange is used to share and extract the initial conditions and the control parameters for the 3D QCLM.

In future work, we will suggest a new deep CNN suitable for generating a digital signature in order to satisfy the identity property. In addition, the integration of the digital signature with the encryption can be designed as a digital deep CNN signcryption scheme. In addition, future research could focus on stream video encryption/decryption, watermarking, data hiding in encrypted images. Moreover, a compression–encryption system based on a new Deep Convolutional Neural Network will be suggested.

## Figures and Tables

**Table 1:** Proposed AEDM NIST Randomization tests.

Tests	*p*-Value	Results
Monobit Frequency	0.4624	Passed
Block Frequency	0.5284	Passed
Runs test	0.3851	Passed
Longest run of ones	0.7253	Passed
Binary matrix rank test	0.2956	Passed
Discrete Fourier transform	0.8542	Passed
Non-overlapping template	0.3825	Passed
Overlapping templates	0.4382	Passed
Universal statistical	0.5831	Passed
Linear complexity	0.6832	Passed
Serial test	0.8426	Passed
Approximate entropy	0.7253	Passed
Cumulative sums (Forward)	0.6152	Passed
Cumulative sums (Revere)	0.3526	Passed
Random excursions	0.3793	Passed
Random excursions variant	0.5916	Passed

**Table 2:** Chi-square test results for 10 encrypted images of different sat. images.

Sat.1 Image	Sat.2 Image	Sat.3 Image
$\mathrm{Mean}\;\chi^{2}$	$\mathrm{Mean}\;\chi^{2}$	$\mathrm{Mean}\;\chi^{2}$
279.6581	273.6581	281.6581

**Table 3:** Chi-square test results for 100 encrypted Lena images under α = 0:05.

Techniqe	Lena	
	$\mathrm{Mean}\;\chi^{2}\;$	$\chi^{2}\;>\;295.2478\;$
Proposed	250.14	3
Ref. [64]	252.53	5
Ref. [65]	264.83	-

**Table 4:** Correlation coefficient values for H, V, and D.

Correlation		H	V	D
Scheme	Image	Cipher	Cipher	Cipher
Proposed	Sat1	0.0007	0.0015	0.0001
	Sat2	0.0034	0.0031	0.0024
	Sat3	−0.0004	−0.0025	−0.0005
	Lena	−0.0002	0.0004	0.0001
Ref. [3]	Lena	0.0004	0.0005	0.0003
Ref. [11]	Lena	−0.0084	−0.0017	−0.0019
Ref. [12]	Lena	−0.0042	0.0004	−0.0036

**Table 5:** Information entropy of cipher images by the proposed algorithm and references.

Image (Lena)	Entropy
Proposed	7.9997
Ref [64]	7.9974
Ref [65]	7.9972
Ref [67]	7.9891

**Table 6:** Key Space Analysis.

Key space size	Proposed	Ref. [11]	Ref. [3]
	2^924^	2^312^	2^284^

**Table 7:** *NPCR* and *UACI* values for Lena Image.

Algorithm	Image Size	Encryption		Authentication
		*NPCR %*	*UACI %*	
**Proposed**	**512 × 512**	99.61	33.47	✓
Ref. [3]	512 × 512	99.61	33.48	**✕**
Ref. [11]	512 × 512	99.61	33.49	**✕**
**Proposed**	**256 × 256**	99.60	33.46	✓
Ref. [13]	256 × 256	99.60	33.46	**✕**
Ref. [14]	256 × 256	99.61	28.61	**✕**

**Table 8:** Peak signal-to-noise ratio for different encrypted images.

Image	Lena	Sat.1	Sat.2	Sat.3
PSNR (dB)	9.4005	8.9716	9.2962	9.1536

**Table 9:** PSNR for Lina image with Gaussian and salt and pepper noise.

Gaussian Noise		Salt and Pepper	
0.001	0.0001	0.01	0.001
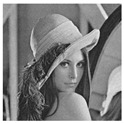	* 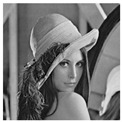 *	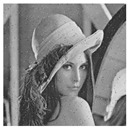	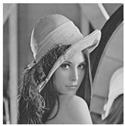
PSNR = 29.7 dB	PSNR = 39.3 dB	PSNR = 25.1 dB	PSNR = 35.4 dB

**Table 10:** PSNR for Lina image with speckle noise and filtering attack.

Speckle		Filtering Attack	
0.01	0.001	(5-5)	(3-3)
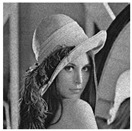	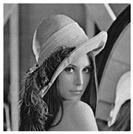	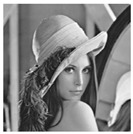	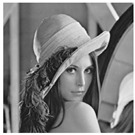
PSNR = 25.3305	PSNR = 35.21 dB	PSNR = 30.44 dB	PSNR = 34.42 dB

## Data Availability

Not applicable.
